# Urinary Incontinence as a Predictor of Death: A Systematic Review and Meta-Analysis

**DOI:** 10.1371/journal.pone.0158992

**Published:** 2016-07-13

**Authors:** Gregor John, Claire Bardini, Christophe Combescure, Patrick Dällenbach

**Affiliations:** 1 Department of Internal medicine, Hôpital neuchâtelois, Chasseral 20, 2300, La Chaux-de-Fonds, Switzerland, Department of Internal Medicine, Rehabilitation and Geriatrics, Geneva University Hospitals (HUG), Gabrielle-Perret-Gentil 4, CH-1205, Geneva, Switzerland; 2 Faculty of Medicine, Geneva University, Geneva, Switzerland; 3 CRC & Division of clinical-epidemiology, Department of health and community medicine, University of Geneva & Geneva University Hospitals (HUG), Geneva, Switzerland; 4 Department of Gynecology and Obstetrics, Perineology Unit, Geneva University Hospitals (HUG), Geneva, Switzerland; National Taiwan University, TAIWAN

## Abstract

**Background:**

The association between urinary incontinence (UI) and increased mortality remains controversial. The objective of our study was to evaluate if this association exists.

**Methods:**

We performed a systematic review and meta-analysis of observational studies comparing death rates among patients suffering from UI to those without incontinence. We searched in Medline, Embase and the Cochrane library using specific keywords. Studies exploring the post-stroke period were excluded. Hazard ratios (HR) were pooled using models with random effects. We stratified UI by gender and by UI severity and pooled all models with adjustment for confounding variables.

**Results:**

Thirty-eight studies were retrieved. When compared to non-urinary incontinent participants, UI was associated with an increase in mortality with pooled non adjusted HR of 2.22 (95%CI 1.77–2.78). The risk increased with UI severity: 1.24 (95%CI: 0.79–1.97) for light, 1.71 (95%CI: 1.26–2.31) for moderate, and 2.72 (95%CI: 1.90–3.87) for severe UI respectively. When pooling adjusted measures of association, the resulting HR was 1.27 (95%CI: 1.13–1.42) and increased progressively for light, moderate and severe UI: 1.07 (95%CI: 0.79–1.44), 1.25 (95%CI: 0.99–1.58), and 1.47 (95%CI: 1.03–2.10) respectively. There was no difference between genders.

**Conclusion:**

UI is a predictor of higher mortality in the general and particularly in the geriatric population. The association increases with the severity of UI and persists when pooling models adjusted for confounders. It is unclear if this association is causative or just reflects an impaired general health condition. As in most meta-analyses of observational studies, methodological issues should be considered when interpreting results.

## Background

Urinary incontinence (UI) -the complaint of any involuntary loss of urine [1]- is frequent in the general population and affects men and women of all ages. It has been found in 11 to 34% of men and 13–50% of women older than 60 years (depending on the method used), and in 43–80% of all nursing home residents [2–4].

UI decreases quality of life of men and women, and has been associated with many unfavourable outcomes, including longer hospital length of stay and lower chance of regaining home after hospital discharge [5–7]. Thus, UI carries an unsuspected load on the healthcare system, with an estimate of 14 billion dollars spent each year in the United States (5 billion dollars for institutionalised citizens), and 4.6 billion Euros in France [8]. Besides, this condition will affect over 423 million people worldwide by 2018 [9].

Frail and older patients are at the highest risk for developing UI [7]. Therefore, mortality rate of patients suffering from UI is expected to be higher than the one of patients who are not suffering from this condition. UI may be a marker of an impaired general health condition and an indirect cause of death consecutive to falls, for example [10]. However the extent of the increased mortality is not clear yet, and varies across studies according to gender, as well as UI severity. Furthermore, after adjustment for the high number of comorbid conditions and disability found among urinary inctontinent patients, studies differ in their conclusions. Some find a persisting association, while others don’t. An association independent of these factors could stimulate research on UI treatment.

Thus the aim of this systematic review and meta-analysis was to determine the effect of UI on mortality, in subgroups of men and women, and according to UI severity strata. We explored all published adjusted models to determine if the association persisted after adjustment for confounders.

## Subjects/Patients and Methods

We performed a systematic review and meta-analysis of all studies exploring UI and death. The study was divided into two subsets: those exploring the post-stroke period (published separately) and those in the general population. Search strategy, study selection, data extraction, and analysis were performed according to a pre-defined protocol (available on request).

### Search strategy

The search strategy in Medline, Embase and the Cochrane library used predefined keywords (Search strategy in S1 File) and was limited to articles written in English, French, and Italian and published before December 7, 2014. We examined reference lists from retrieved articles, guidelines and systematic reviews and asked experts in urology and gynaecology for studies we might have missed.

### Study selection and data extraction

We included retrospective and prospective studies comparing mortality rate between patients with and without UI. Urge, stress, or mixed UI had not to be the main focus of the study. Diurnal and nocturnal episodes of UI that happened at least once during the previous year were considered. UI could be diagnosed based either on caregiver records or patient’s self reporting, and as defined according to the International Continence Society by the complaint of any involuntary loss of urine [1]. Publications on the same cohort of patients were all included, but duplicate data was avoided in the meta-analyses. We excluded all case reports, studies with patients under eighteen, and articles exploring the post-stroke period.

CB and GJ independently evaluated studies for possible inclusion. Irrelevant studies were excluded based on title and abstract. Full texts were then obtained to ascertain each study’s eligibility and for data extraction. Variables extracted in all studies were: all-cause mortality of patients with and without UI, study design, population characteristics, place of living (or inclusion), variables used in adjusted models, and UI sub-type, severity, and definition. Disagreements on article inclusion and data extraction were resolved by consensus.

### Quality assessment

The quality of the observational cohort studies was assessed through the Newcastle-Ottawa Quality Assessment scale (NOS) [11]. This scale explores three domains: "selection of study groups", "comparability of groups", and "ascertainment of exposure/outcome". In the "selection of study groups" domain, two points were automatically achieved in all studies ("demonstration that outcome of interest was not present at start of study" and "selection of the non exposed cohort"), one point was obtained if the cohort was not restricted to a specific sub-population (eg. post fracture), and another point if UI was diagnosed through a valid structured questionnaire. One point was given to the domain "comparability of groups" if the analyses were adjusted for age and two points if another adjustment variable was used in the models. Regarding the "ascertainment of exposure/outcome" domain, one point was attributed if the follow-up was more than one month, a second point if subjects lost were less than 10% (or if a description was provided for all patients lost to follow-up) and a third point for the assessment of outcome. Two investigators (CB, GJ) assessed study quality independently.

### Pooled data analysis

Measures of association were unadjusted and adjusted hazard ratios (HR), between UI and mortality. When several HRs were reported, we kept the HR for the longest follow-up period. If published information was not sufficient to extract HR, authors were contacted. If the HR could not be obtained, we estimated it and its variance using the ratio of logarithms of event-free proportions from the proportion of death in the exposed and unexposed groups. [12] This method uses the published proportion of death, and does not consider patients lost to follow-up.

For one study [13], the unadjusted HRs were derived from the published survival curves: survival estimates were extracted from the digitalized picture of survival curves. Individual survival data were extrapolated from the sample size and the survival estimates. The estimate of HR was then obtained using a Cox regression model. The censorship rate was very low in this study and we assumed a null censorship rate to derive the HRs.

In two studies adjusted HRs were only given by gender. We assessed a global logarithm of HR as a weighted average of the gender-specific logarithm of HRs (Supplemental statistics A in S1 File). Three studies reported the ajusted HR by sub-group of severity of UI but no global HR for UI. We combined the HRs reported by severity of UI in a single global HR using a similar approach. However, in contrast to the gender-specific HRs, the severity-specific HRs from a study are not independent, as the same control participants (continent subjects) are used to calculate the HR in the different UI severity strata. Therefore, we pooled the severity-specific HRs assuming a correlation between the HRs of 0.20 (Supplemental statistics A in S1 File). The validity of this approach was tested by re-assessing global HRs–through this approach- for all studies and comparing them to the published global HR. We then explored the effect of varying the coefficient of correlation of the three considered studies on the pool estimates of adjusted HRs. The result was robust (Supplemental statistics A in S1 File).

All pooled estimates (except for those dedicated to summarize HRs for a single study) were systematically obtained using models with random effects (Der Simonian and Laird’s method). The significance level was set at 0.05 for all analyses and all statistical tests were two-sided.

We performed other meta-analyses to explore a modification of the association between UI and mortality by gender and severity of UI. For gender, we pooled the difference in logarithm of HR between men and women. By taking the exponential, we expressed results as an increase/decrease of HR in men compared with women. A positive difference means that the association between UI and mortality is stronger in men than in women. With this approach, the advantage is that we account for the fact that HR in men and women are assessed in the same studies.

For UI severity, we used two strategies. First, we stratified severity using the categories published in any articles regardless of the differences in definitions between studies. Second, we classified all articles (and severity strata, when available in articles) depending on the least number of episodes of urinary leakage needed for the diagnosis of UI. Thus we grouped monthly, weekly, or daily episodes of UI. To analyse the increase of HR with the severity of UI, we assessed for each study the difference in logarithm of HRs between two stages of severity (assuming a correlation of 0.20) and we combined these differences (Supplemental statistics B in S1 File). The results were expressed as the pooled ratio of HRs between two stages of severity of UI (exponential of the pooled difference in logarithm of HRs).

Heterogeneity was measured with I^2^ statistics (>75% being considered highly heterogeneous). Potential heterogeneity factors were explored by leave-one-out strategy, and sub-group analyses. Pre-specified subgroup analysis included: stratification by study design, date of publication, country, population studied (general geriatrics versus specific population), and settings. Settings were divided in community, hospital, nursing care, and a mixture of these. We stratified the pooled adjusted models into two categories: highly adjusted models (with adjustment for at least both functional status and age) and models with low adjustment variables. We tested heterogeneity across subgroups. [14]

In sensitivity analyses, we pooled published odds ratios (OR) stratified by length of follow-up (6 months, 1, 3, 5, and 10 years). We also restricted the analysis to the studies with good to fair quality in each of the three domains of the NOS.

Publication bias for each outcome was graphically explored through funnel plot and Egger's test. The trim and fill method was used to check the impact of a potential publication bias on pooled estimates. The R package “meta: Meta analysis with R”, version 1.6–1 and Review Manager of the Cochrane Library (RevMan), version 5.3 was used for these analyses.

## Results

**Fig 1:**
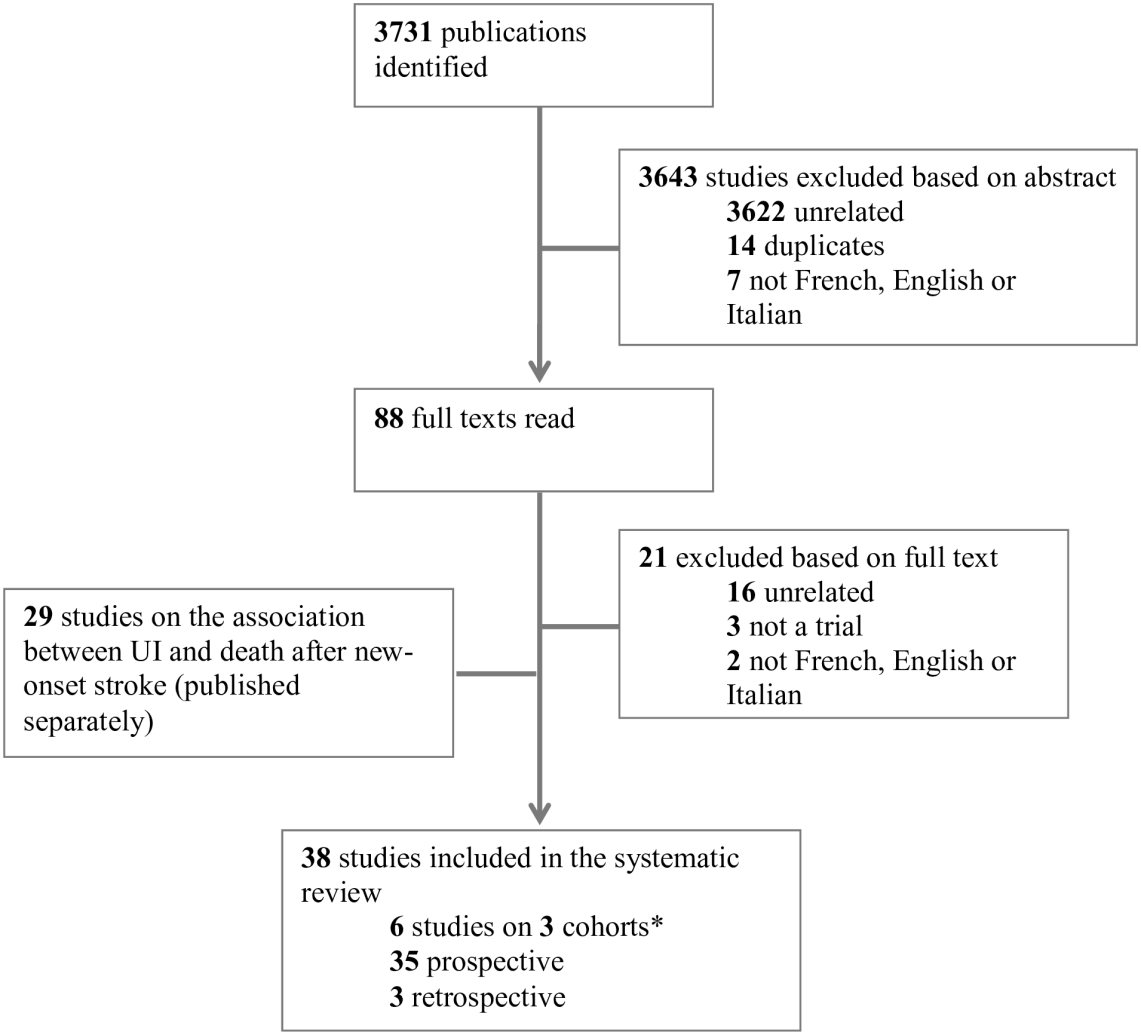
Flow chart of identified references to retrieved studies in the systematic review. *Three longitudinal cohorts gave multiple publications on the association between urinary incontinence and mortality, at different time points.

We explored 3731 citations and retrieved 38 studies (exploring 35 single cohorts) (Fig 1). Six studies reported association between UI and death at different time points at follow-up for 3 individual cohorts. The main characteristics of the studies included in the systematic review are displayed in Table 1.

**Table 1:** Main characteristics of the studies included in the review.

**First author/ year**	**Design**	**Country**	**N**	**Follow-up (m)**	**Age**	**UI (%)**	**Patient**	**Inclusion**	**Men (%)**	**Death (%)**		**Association**		**Other association**
										**UI**	**Controls**	**Un-adj**	**Adj**	
Anpalahan 2008 [15]	prosp	Australia	110	3	83	16.4	Geriatrics	Hospital	31.8	33.3	3.3	+ *	+	HC(+), read(+), LOS(+)
Adams 2000 [16]	prosp	France	45	48	41	20	Fam amyl PNP	Community	55.5	30.0	8.6	+	+	-
Abrahamik 1993 [17]	prosp	France	1025	2	78.1	41%	Geriatrics	Hospital	43.5	20.2	10.1	+	NA	IUC(+), LOS(+)
Baztan 2005 [18]	prosp	Spain	205	6	80	68.6	Geriatrics	Hospital	39.5	14.9	3.1	+	+	disability(+)
Berrios 1986 [19]	prosp	UK	100	18	80.5	35	Cognitive failure	Community	40.8	68.6	35.4	+	NA	-
Berardelli 2013 [20]	prosp	Italy	570	84	73 + 92^†^	32.1	Geriatrics	Community	43.3	-	-	+/-^‡^	+/-^‡^	Frailty(+)
Bootsma 2013 [21]	prosp	Netherlands	639	12	78.2	20.7	Geriatrics	Hospital	46.2	36.4	31.3	+/-^§^	-	HC(+), disability(+)
Brauer 1978 [22]	prosp	Denmark	1486	24	80–89^||^	NA	Geriatrics	HC	34.7	-	-	+	+	-
Campbell 1985^¶^ [23]	prosp	New Zealand	559	36	80–84^||^	18.7	Geriatrics	Community /HC	35.2	72.5	34.6	+	+	-
Campbell 1985^¶^ [24]														
Chen 2010 [25]	prosp	Taiwan	559	12	80.9	6.8	Geriatrics	HC	100	18.4	8.2	+*	+*	-
Donaldson 1983^¶^ [26]	prosp	UK	4490	36	75–84^||^	NA	Geriatrics	Hospital/HC	NA	52.7	42.7	+	+	-
Donaldson 1980^¶^ [27]			4514	12									NA	
Ekelund 1987 [28]	prosp	Sweden	837	6	NA	27.9	Geriatrics	Hospital	37.6	36.9	19.5	+	NA	HC(+)
Espallargues 2008 [29]	prosp	6 countries	1667	1	78.1	18.5	Geriatrics	Hospital	43.5	-	-	+ *	- *	HC(+), read(+), LOS(+)
Gambassi 1999 [30]	prosp	USA	9264	23	82.1	60.5	Alzheimer	HC	30.8	55.5	41.4	+	+/-**	-
Gavira 2005 [31]	prosp	Spain	827	60	75–84^||^	39.8	Geriatrics	Community	41.2	23.0	20.7	-	-	-
Goldfarb 1969 [32]	prosp	USA	1280	84	75–84^||^	21	Geriatrics	HC	33.3	97.1	78.0	+	NA	-
Herzog 1994 [33]	prosp	USA	1956	72	60–69^||^	29.9	Geriatrics	Community	41.1	19.7	21.7	-	-	-
Hollins 1998 [34]	prosp	UK	2026	96	NA	39.9	Learning disability	Community/ HC	NA	21.1	8.1	+	+	-
Holroyd-Leduc 2004 [35]	prosp	USA	6506	24	77	14.8	Geriatrics	Community	37	10.9	8.7	+	-	HC(-), disability(+)
**First author/ year**	**Design**	**Country**	**N**	**Follow-up (m)**	**Age**	**UI (%)**	**Patient**	**Inclusion**	**Men (%)**	**Death (%) for**		**Association**		**Other association**
										**UI**	**Controls**	**Unadj**	**Adj**	
John 2014 [6]	retro	Switzerland	699	36	80	27.8	Home care services	Community	24.6	24.9	12.8	+	+	HC(-), read(-) LOS(+)
Johnson 2000 [13]	prosp	USA	3485	36	75–84^||^	28.7	Geriatrics	Community	51.5	-	-	+	+/-^§§^	-
Kohn 1991 [36]	prosp	Israel	188	60	82.2	30.1	Geriatrics	Hospital	42.1	95.9	70.8	+	NA	-
Koyano 1986 [37]	prosp	Japan	2567	60	72.4	8.8	Geriatrics	Community	47.6	57.2	15.4	+	NA	disability(+)
Krumholz 2001 [20]	prosp	USA	103164	12	76.8	22.7	Myocardial infarct	Hospital	50.1	45.7	14.6	+	+	-
Landi 2012 [38]	prosp	Italy	2787	12	80.4	54.9	Geriatrics	Community	39.8	-	-	+	NA	Low BMI(+)
Luk 2013 [39]	prosp	Hong Kong	312	12	88	99	Cognitive failure	HC	22.8	34.3	33.3	-	NA	-
Min 2009 [40]	prosp	USA	649	60	82	35.9	Geriatrics	Community	37.2	-	-	+	NA	-
Nakanishi 1999 [41]	prosp	Japan	1405	42	65–74^||^	11.9	Geriatrics	Community	40.1	35.3	11.1	+^‡‡^	+^‡‡^	-
Nuotio 2009^¶^ [42]	prosp	Finland	398	72	70–79^||^	31.9	Geriatrics	Community /HC	43.5	42.5	25.9	+	-	-
Nuotio 2002^¶^ [43]			1052	120	73.3	5.6			49.8	78.0	47.8	+	+/-^††^	-
Pagliacci 2007 [44]	prosp	Italy	511	48	41.9	NA	Spinal cord injury	Hospital	80	-	-	+^‡‡^	+^‡‡^	Read(+),LOS(+) complication(+)
Sorbye 2013 [45]	prosp	Norway	331	12	84.2	49.2	Hip fracture	Hospital	20.2	20.2	10.1	+	NA	HC (+), disability (+), IUC(+), fall(+)
Thom 1997 [5]	retro	USA	5986	108	75–79^||^	6.1	Geriatrics	Community	49.8	40.9	25.5	+	+/-^††^	HC (+), read(+)
Tilvis 1995 [46]	prosp	Finland	649	60	79.7	19.3	Geriatrics	Hospital/HC	26.3	39.5	22.7	+	-	HC (-)
Venkatsen 1990 [22]	prosp	UK	73	1.5	79	7	Pneumonia >65y	Hospital	52.1	-	-	+	NA	-
Zweig 1990 [21]	retro	USA	133	1	80	44.4	Pneumonia >60y	Hospital	45	20.3	12.2	+	-	-

* Urinary incontinence along with other geriatric symptoms.

^†^ Two cohorts;

^‡^ association found among sever UI;

^§^ association found at three month not 12 month;

^||^ mode;

^¶^ same cohort published in two articles;

** only for moderate dementia;

^††^ association found for men, not women;

^‡‡^ for bowel and urinary loss of control.

^§§^ depending on the adjusted model considerate.

BMI: body mass index; fam amyl PNP: familial amyloidoic polyneuropathy; HC: home care; read: hospital readmission; IUC: indwelling urinary catheters; LOS: length of hospital stay; NA: not assessed; UI: urinary incontinence; unadj and adj: association between urinary incontinence and death unadjusted or adjusted for confounders; prosp: prospective study; retro: retrospective study; read: hospital readmission; >65y: patients older than 65 years old.

This review included 158 456 patients from nineteen countries. The prevalence of UI ranged from 5.6% to 99%, but the proportion of incontinent patients was unknown in four studies [17, 22,44,47]. The selected studies mainly explored the effect of UI in the general geriatric population. Nine articles assessed this effect on more specific populations such as individuals affected by cognitive failure [19], Alzheimer’s disease [30], spinal cord injury [44], post myocardial infarction [20], learning disability [34], pneumonia [21,22], post surgery after a hip fracture [45] and familial amyloid polyneuropathy after liver transplantation [16]. Time to follow-up ranged from four weeks [21,29] to ten years [43]. Twelve studies included hospitalized patients, 13 explored participants in the community, five among home care patients, and five studies among a mixture of settings (hospitalized and home care patients [26,46] or community and home care [23,34,42]).

Seven studies [15,22,29,38,40,44,47] included in the review could not be incorporated in the meta-analysis as part of the data on UI and mortality was lacking. The authors of those studies were unreachable or the databases were no longer available.

### Unadjusted association between UI and death

All but three [31,33,48] out of 38 studies (92.1%) found a positive association between UI and death in unadjusted analysis. However some studies considered the association between death and UI along with other geriatric symptoms [15,25,29] or together with faecal incontinence [41,44]. The association was true only for severe UI in the study by Berardelli et al., and only found at three months (not afterwards) in the study by Bootsma et al.

**Fig 2:**
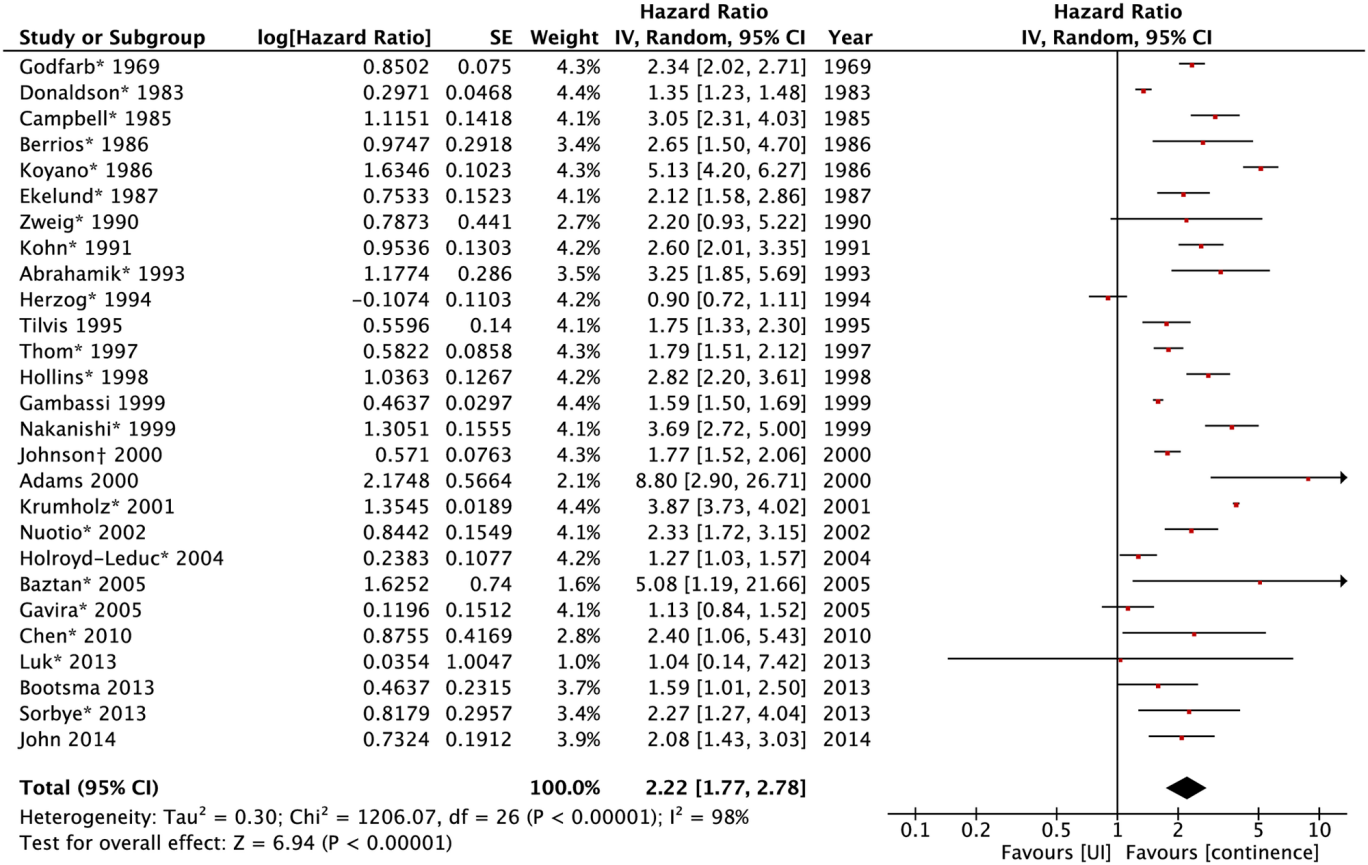
Forest plot of unadjusted HR of death for urinary incontinence. * Estimated from the ratio of logarithms of event-free proportions from the published proportion of death in the exposed and unexposed groups. † HR could be estimated from the Kaplan-Meier curve for one study UI: urinary incontinence.

Unadjusted survival analysis was available in 5 studies (Fig 2). Estimated HR from published proportions of death was calculated for 21 studies. HR could be estimated from the Kaplan-Meier curve for one study [13]. The resulting pooled HR was 2.22 (95%CI: 1.77–2.78).

In a sensitivity analysis, a pooled analysis was done computing all OR published, stratified by their follow-up period (Fig A in S2 File). The result was statistically significant for all periods.

**Fig 3:**
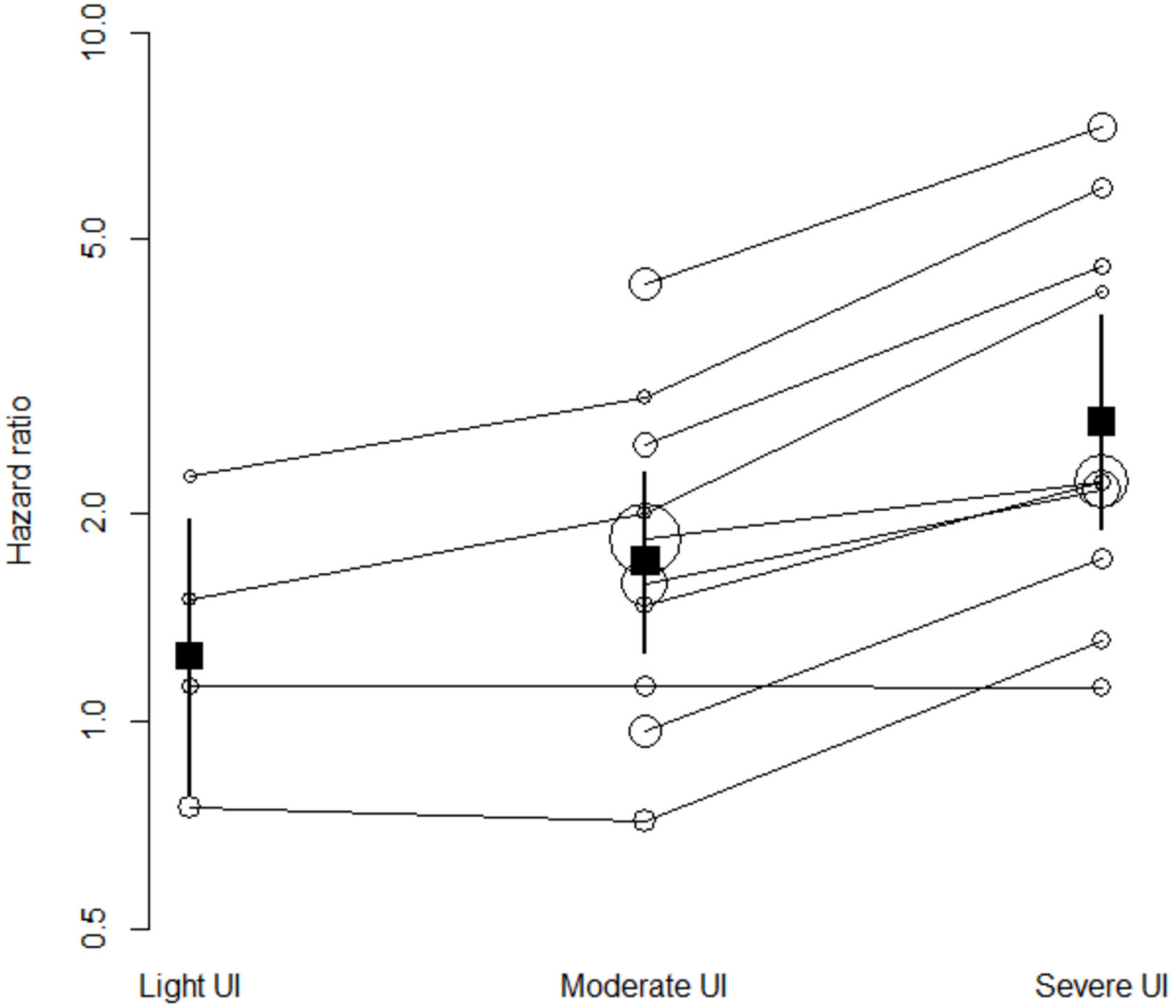
Unadjusted HRs of studies (white circles) and pooled HRs (black boxes) of death for urinary incontinence by (published) UI severity. Circle-sizes are inversely proportional to studies' standard error. UI severity subgroups are bounded by solid lines. UI: urinary incontinence.

The association between UI and time to death increased gradually with the severity of leakage, when stratified according by published categories (light, moderate, severe) (Fig 3) or extracted frequencies of leakage episodes (Fig B in S2 File). In the analyses conducted with all studies, the pooled HRs were 1.24 (95%CI 0.79–1.97; I^2^ = 71%), 1.71 (95%CI 1.26–2.31; I^2^ = 92%), and 2.72 (95%CI 1.90–3.87; I^2^ = 92%) for light, moderate, and severe UI respectively (Fig 3). Four studies reported HRs for both light and severe UI. The pooled ratio of HRs was 1.83 (95%CI 1.16–2.89). Ten studies reported HRs for both moderate and severe UI. The pooled ratio of HRs was 1.47 (95%CI 1.28–1.69). Thus the HR in patients with severe UI was approximately 1.8 times the HR in patients with light UI and 1.5 times the HR in patients with moderate UI and those differences were statistically significant. Four studies reported HRs for both light and moderate UI. The pooled ratio of HRs was 1.08 (95%CI: 0.81–1.44): the HR was slightly greater in patients with moderate UI than in patients with light UI, but the difference was not statistically significant.

In seven studies, association between UI and death could be stratified by gender and another study included only men [25]. Although the HR was slightly higher for men 2.23 (95%CI: 1.45–3.42) compared to women 2.01 (95%CI: 1.19–3.38), there was no statistical difference in the logarithmic of HR between genders (Fig C in S2 File).

### Adjusted association between UI and death

**Fig 4:**
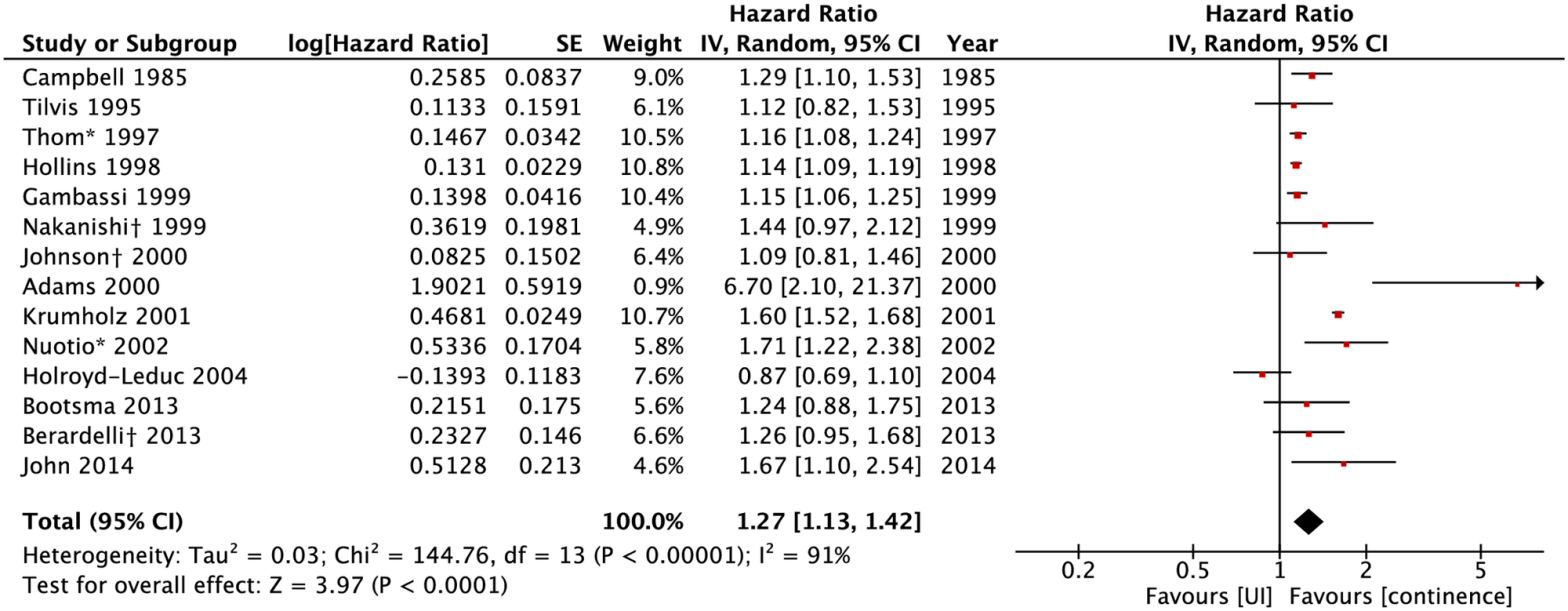
Forest plot of adjusted HR of death for urinary incontinence. Adjusted HR was published by gender (*) or severity of UI subgroups (†) only. A summarized HR was obtained through a meta-analysis of all subgroups for each of those studies. UI: urinary incontinence.

Twelve studies (31.6%) included in the review gave no adjusted results and eight (21.0%) showed no association between UI and death after adjustment for different confounders. Sixteen studies (42.2%) still showed a positive association in adjusted models (Table 1). Adjusted HRs were available in fourteen studies (Fig 4). UI was associated with death with a pooled HR of 1.27 (95%CI: 1.13–1.42).

**Fig 5:**
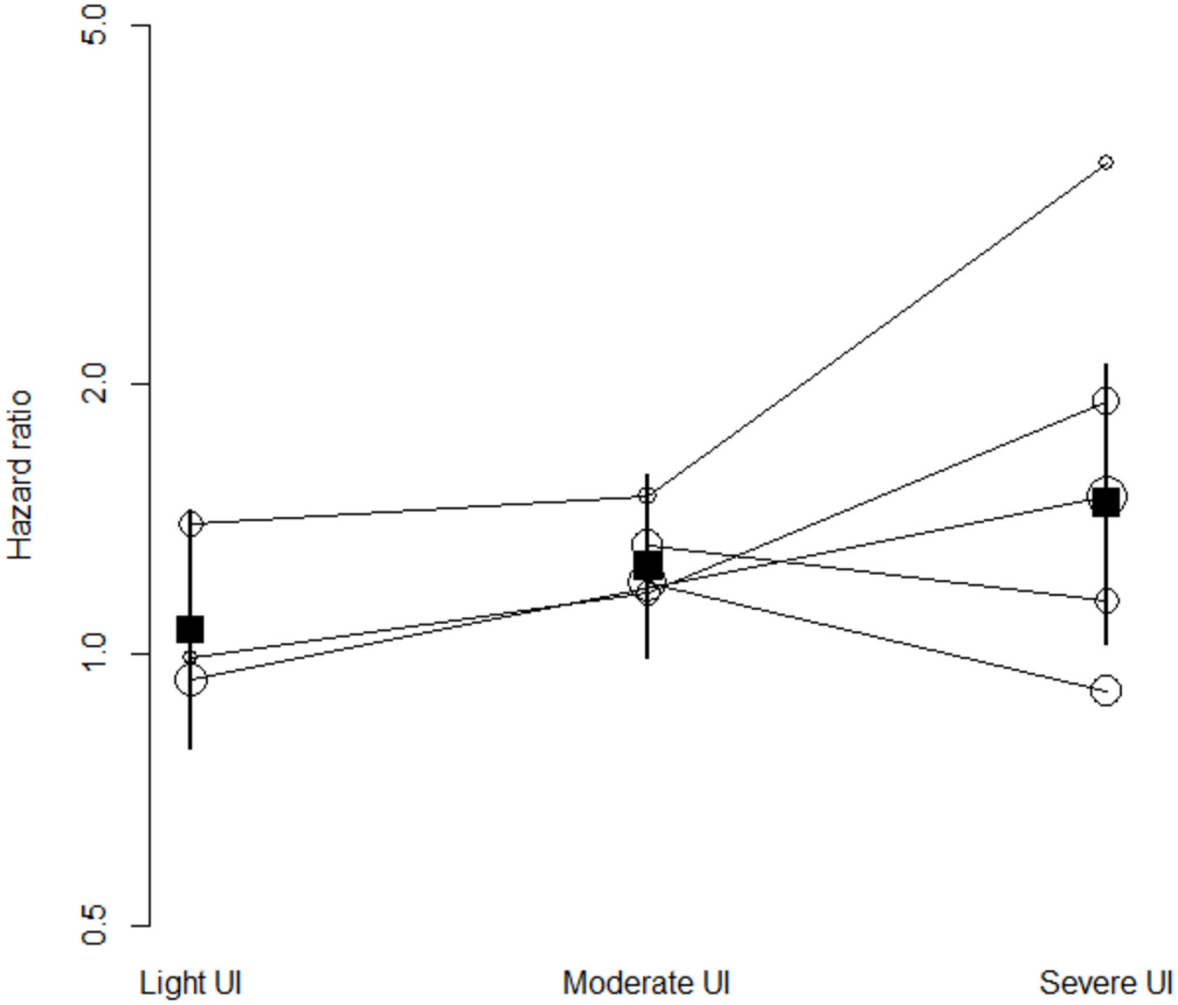
Adjusted HRs (white circles) and pooled HRs (black boxes) of death for urinary incontinence by (published) UI severity. Circle-sizes are inversely proportional to studies' standard error. UI severity subgroups are bounded by solid lines. UI: urinary incontinence.

The association between UI and time to death increased gradually with the severity of leakage, when stratified according to published categories (Fig 5), or estimated frequencies of leakage episodes (Fig D in S2 File). In the analyses conducted with all studies, the pooled HRs were 1.07 (95%CI 0.79–1.44; I^2^ = 0%), 1.25 (95%CI 0.99–1.58; I^2^ = 0%), and 1.47 (95%CI 1.03–2.10; I^2^ = 61%) for light, moderate, and severe UI respectively (Fig 5). Three studies reported HRs for both light and severe UI. The pooled ratio of HRs was 1.79 (95%CI 1.23–2.61): the HR in patients with severe UI was approximately 1.8 times the HR in patients with light UI and the difference was statistically significant. Four studies reported HRs for both moderate and severe UI and two studies reported HRs for both light and moderate UI. The pooled ratio of HRs were 1.12 (95%CI 0.71–1.75) and 1.13 (95%CI 0.63–2.01), respectively: the HR was slightly greater in patients with severe UI than in patients with moderate UI, and in patients with moderate UI compared to patients with light UI, but those differences were not statistically significant.

In three studies, association between UI and death could be stratified by gender. The HRs for men and women were 1.50 (95%CI: 1.01–2.22) and 1.17 (95%CI: 1.00–1.37), respectively. There was no statistical difference between genders (Fig E in S2 File).

### Study quality/risk of bias

#### Quality scale

The NOS is shown in Table A in S2 File. Quality was mainly limited by poor definition of exposure (UI) and comparability of groups. Many studies defined UI based on unspecified personal patient information, carer report [27,34,46,47,49], or medical records [5,20,21,26]. Only 19 reports were based on more reliable sources like specific questionnaires [41,42,50,13,17,40] or pre-existing scales such as the modified Barthel index [18,39], or the Minimum Data Set [6,30,38,45]. Information on the diagnosis of UI was lacking in many articles. In eight studies patients with indwelling urinary catheters were classified as being incontinent of urine [6,17,18,23,24,31,45,50]. However, except for five studies [13,15,28,39,49], most of the other studies gave no information regarding that consideration. Comparability was limited when none or low adjustment was reported.

#### Sources of heterogeneity

The results of stratified subgroup analysis are shown in Table 2. No statistically significant differences were found between subgroups that were observed both in unadjusted and adjusted models. For the unadjusted pool analysis, the country where the study took place explained part of the heterogeneity. In unadjusted models, the published HR subgroup represented 23.8% of the total population and had only mild heterogeneity (I^2^ 48%). The variables of adjustment included in the models varied greatly, but could be regrouped into categories (Table B in S2 File). The high heterogeneity found in unadjusted models (I^2^ 98%) decreased marginally in adjusted ones (I^2^ 88%), particularly in the subgroup of highly adjusted models (I^2^ 47%), but the subgroup difference was not statistically different.

**Table 2:** Subgroup analyses.

	Unadjusted analysis				Adjusted analysis			
Factors	N studies	Pooled HR	Within strata	Between strata comparison	N studies	Pooled HR	Within strata	Between strata comparison
***Publication year***								
Less than 10 y	7	1.77 (1.29 to 2.42)	0.0004		3	1.34 (1.08 to 1.65)	0.0070	
10 to 20 y	10	2.29 (1.59 to 3.30)	<0.0001	0.45	9	1.26 (1.09 to 1.46)	0.0020	0.88
More than 20 y	10	2.30 (1.61 to 3.29)	<0.0001		2	1.25 (1.09 to 1.45)	0.0002	
***Study' continent***								
America (North)	7	1.83 (1.16 to 2.91)	<0.0001	0.02	5	1.17 (0.96 to 1.43)	0.1300	0.56
Asia/Oceania	6	3.31 (2.44 to 4.49)	<0.0001		2	1.32 (1.13 to 1.53)	0.0003	
Europe	15	2.07 (1.75 to 2.45)	<0.0001		7	1.35 (1.11 to 1.65)	0.0020	
***Population studied***								
General geriatrics	18	2.06 (1.66 to 2.56)	<0.0001	0.42	9	1.19 (1.07 to 1.32)	0.0010	0.19
Other	9	2.53 (1.62 to 3.97)	<0.0001		5	1.40 (1.12 to 1.75)	0.0030	
***Setting***								
Hospital inpatients	8	2.60 (1.92 to 3.52)	<0.0001		2	1.49 (1.20 to 1.86)	0.0003	
Community	10	2.07 (1.43 to 3.00)	<0.0001		7	1.23 (1.01 to 1.49)	0.0400	
Home care	4	1.94 (1.39 to 2.71)	<0.0001	0.61	1	1.15 (1.06 to 1.25)	0.0008	0.16
Mix	5	2.14 (1.47 to 3.13)	<0.0001		4	1.24 (1.08 to 1.43)	0.0030	
***Follow-up***								
<1 y	4	2.38 (1.85 to 3.05)	<0.0001		0	-	-	
1–5 y	14	2.21 (1.57 to 3.10)	<0.0001	0.79	10	1.30 (1.09 to 1.55)	0.0040	0.23
>5 y	9	2.06 (1.47 to 2.88)	<0.0001		4	1.16 (1.10 to 1.23)	<0.0001	
***Design***								
*Prospective*	13	2.20 (1.71 to 2.82)	<0.0001	0.68	12	1.26 (1.10 to 1.45)	0.0009	0.85
*Retrospective*	3	2.05 (1.63 to 2.57)	<0.0001		2	1.31 (0.94 to 1.84)	0.1200	
Adjustment level*								
Low	-	-	-	-	6	1.31 (1.09 to 1.57)	0.0040	0.54
High	-	-	-		8	1.22 (1.06 to 1.40)	0.0070	

* Highly adjusted models are those with adjustment for at least both functional status and neurological deficit.

HR: hazard ratio; NOQ scale: Newcastle-Ottawa Quality Assessment scale; UIC: urinary indwelling catheters; y: years

#### Risk of publication bias

**Fig 6:**
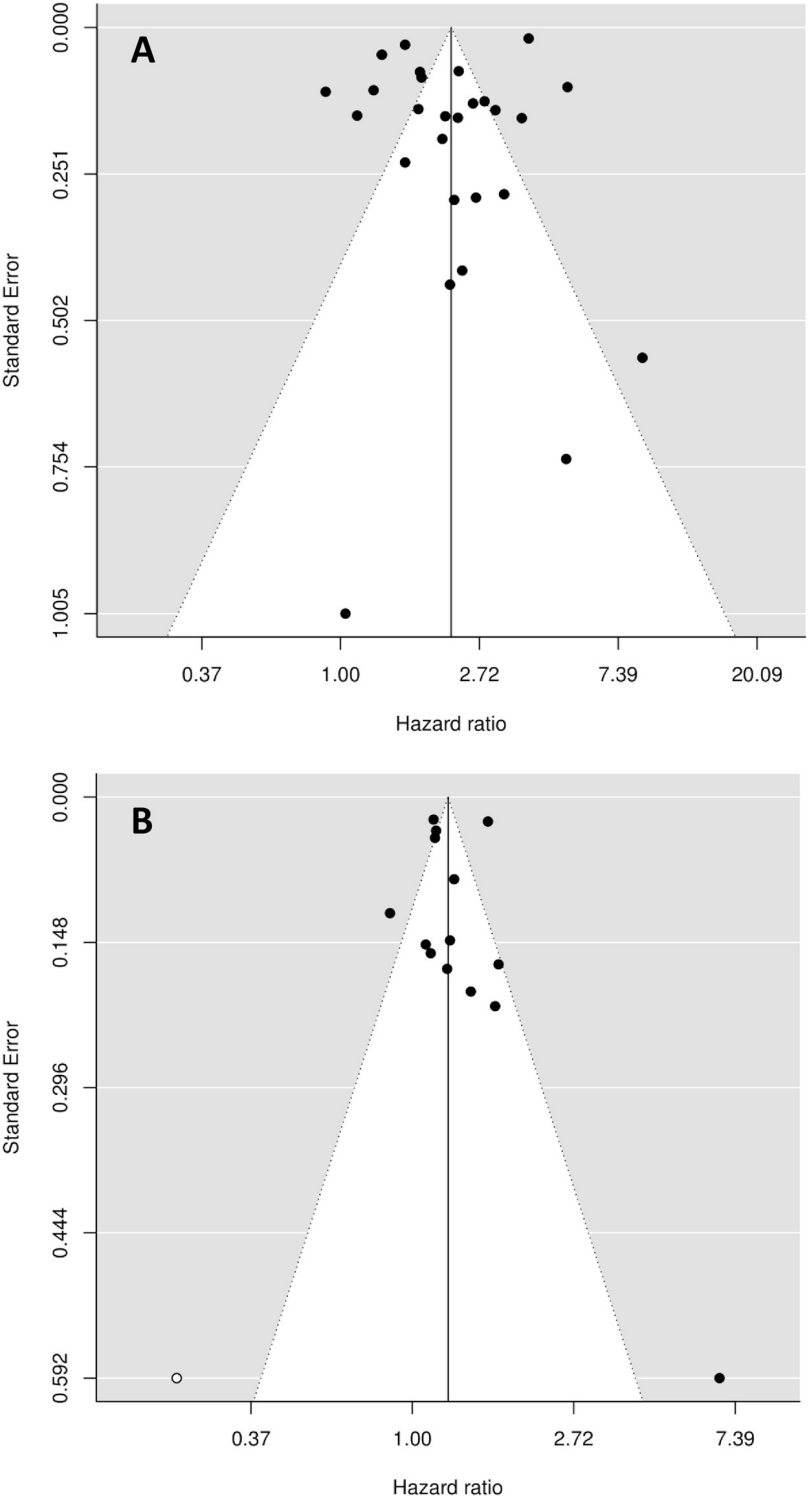
Funnel plot for unadjusted (panel A) or adjusted (panel B) HR of death. Panel A: With The Trim and Fill approach, no missing study was detected. Panel B: With The Trim and Fill approach, one missing study was detected on the left part of the funnel plot (white dot).

Funnel plots showed no obvious publication bias and Egger test failed to detect heterogeneity (Fig 6). However, for the adjusted association between UI and death, the Fill and Trim method detected one missing study on the left part of the funnel plot. When adding this hypothetical study, the pooled HR was not affected: 1.25 (95%CI 1.11–1.40).

#### Sensitivity analyses

The pooled published ORs gave the same results as the pooled estimated HRs. Taking out all studies one by one did not alter the direction of the association. However in adjusted analysis, the study by Krumholz et al. explained part of the heterogeneity as the I^2^ decreased from 91% to 55% without changing the extent of the association. This large-scale prospective study explores a specific population of elderly patients after heart infarct.

When restricting the analysis to studies with good to fair quality in each of the three domains of the NOS, no differences were noted for most of the adjusted and unadjusted pool estimates (Table C in S2 File). However, the pooled unadjusted HR dropped to 1.85 (95%CI: 1.33–2.58), when studies with poor quality in the domain of “comparability of groups” were excluded. When studies with poor quality in the domain of “selection of study groups" were excluded, the pooled adjusted HR was 1.17 (95%CI: 1.04–1.32).

## Discussion

Our study confirms that UI is associated with a higher risk of death in the general (geriatric) population. The association is also seen in a broad range of specific conditions (e.g.: myocardial infarct, hip fracture, or cognitive impairment) and in all settings (hospitalized patients, nursing home residents, and patients living in the community). The risk increases with the severity and number of events of urinary leakage, exists for men and women, and persists in adjusted survival regression models.

Only four studies retrieved in this systematic review gave the cause of death and no assumption could be made for a different cause in the subgroup of UI patients [16,22,34,39]. Pneumonia represented 20–65% of the deaths. However these studies were not representative of the general population and death might be bound to the disease/condition itself (eg infection after immunosuppressant [16], or pneumonia for patients with neurologic impairment [22,34,39]).

The association between UI and death is probably multifactorial. On one hand, risk factors for the development of UI by themselves have a negative impact on survival. Indeed this meta-analysis shows that the association is closely tied to age, comorbid conditions, and disability, since the pooled HR of 2.2 is reduced to 1.3 when pooling adjusted models. For this reason, unadjusted HR should not be interpreted on its own and studies exploring UI and death should report adjusted HR. On the other hand, UI gives rise to multiple unfavourable outcomes [7], such as increased risk of falls and related injuries [10,51,52], depression [53], and infections [54–56]. Infections affect 20% of patients with UI and cause a mortality rate of 0.3%. Falls and depression increase mortality by 15% and 17% respectively [57]. However, the exact interaction between fall, depression, infection, and UI is difficult to assess and must have multiple interconnections, since all of those symptoms are frequent in the general elderly population and share many confounding factors. Thus, mortality is probably not entirely explained by those conditions.

New evidence and understanding of the pathophysiology of UI have gone way beyond the simple “mechanical” model of UI. Higher intakes of some micronutrients such as calcium [58], vitamin B12, and Zinc [59], as well as the total energy consumption, and saturated (opposed to polyunsaturated) fat are associated with UI [59,60]. Indeed, the most promising gene associated with lower urinary tract symptoms is a variant of the vitamin D receptor [61]. Vitamin D and calcium have both been extensively studied and associated with death [62–64]. Finally, recurrent infections, and/or a specific microbiota in the bladder of UI patients [65–67] might trigger a systemic mechanism. The genetics, the microbiota, and the nutritional theories are promising and could offer a perspective for future research to find the missing rational link between UI and death.

A causal association is supported by the dose response observed across studies. Nevertheless, meta-analysis of observational studies always face methodological limits. The remaining effect after adjustment for confounding factors (like age, disability and comorbid conditions) may be explained by a persisting confounding effect (under adjustment), or a publication bias (only significant adjusted models, or only models where the UI is associated with death are published). In favour of the last two hypotheses is the fact that the effect of published models is close to each other, independently of the number of the variables used to adjust. Published models would be those with a maximum covariate and persistent positive effect (or with only a small loss of statistical significance). Furthermore, not all adjusted models included all-important confounding factors. To overcome this hypothesis would necessitate a meta-analysis of individual data and/or to adjust all models with the same confounding factors. The second argument for bias unrecognized by standard evaluation is the fact that one third of studies gave no adjusted models.

To our knowledge, there are no studies exploring specific UI treatments with drugs or surgery using mortality as the main outcome. Future studies exploring the decreased mortality after UI treatment would strengthen the causal hypothesis. A relative drawback comes from the fact that some interventions to reduce UI (weight loss/bariatric surgery or treatments addressing the disability) are also prone to affect mortality [68,69]. Causal or not, the association between UI and death is strong, and could offer–by simply UI and its severity assessment- an overall mortality risk indicator. Unfortunately, UI is still often overlooked with only around half of UI patients seeking medical help [70].

The strength of our study is the use of HR (reported, calculated or estimated) as an effect estimate to pool results. Many published models report ORs, but this measure of association overestimates the real ratio of incidence, especially when the event is frequent (more than 10%) or follow-up is long, and is limited to the specific time-point considered (e.g. at 1 month post inclusion) [12]. The two strategies of UI severity stratification make the dose-response more reliable. Nevertheless, this meta-analysis has several limitations. Firstly, the definition of UI used is inconsistent across studies and often based on unreliable sources (eg medical chart or not specified). Secondly, most pooled HRs were estimated from the proportion of deaths. This measure does not take into account the loss to follow-up. However, the sensitivity analysis pooling published OR from a given proportion of deaths gave similar results. Thirdly, we could not stratify the analyses on UI subtype (urge, stress and mixed type incontinence), which have different risk factors and possibly different impacts on mortality.

## Conclusion

UI is a predictor of higher mortality in the general and particularly in the geriatric population. The association increases with the severity of UI and persists when pooling models adjusted for confounders. UI being a widely spread disorder, more attention should be given to the elderly in terms of its screening and treatment.

## Supporting Information

S1 File Search strategy. Supplemental statistics A: assessment of global HR from different subgroups. Supplemental statistics B: pooled HR ratios for UI severity. (DOCX)

S2 File Table A. Newcastle-Ottawa Quality Assessment scale. Table B. Adjustment variables included in multivariate models. Table C. Subgroup analyses stratified on the three domains of the Newcastle-Ottawa Quality Assessment scale. Table D. PRISMA checklist. Fig A. Forest plot of adjusted OR of death at 6 months, 1, 3, 5, and 10 years. Fig B. Forest plot of unadjusted HR of death for urinary incontinence by frequencies of leakage episodes. Fig C. Forest plot of unadjusted difference of logarithm of HR between men and women. Fig D. Forest plot of adjusted HR of death for urinary incontinence by frequencies of leakage episodes. Fig E. Forest plot of adjusted difference of logarithm of HR between men and women. (DOCX)

## Abbreviations

HR: hazard ratio
NOS: the Newcastle-Ottawa Quality Assessment scale
OR: odds ratio
UI: urinary incontinence
