# Supplementary material for: Urinary Incontinence as a Predictor of Death: A Systematic Review and Meta-Analysis
Source: PLoS One. 2016 Jul 13;11(7):e0158992. doi: 10.1371/journal.pone.0158992 (PMC4943733; doi:10.1371/journal.pone.0158992)

Supplemental material S1

Supplemental Methods 1: Search strategy p. 2

Supplemental Methods 2: Assessment of global HR from different subgroups p. 3

Supplemental Methods 3: Pooled HR ratios for UI severity p. 5

**Supplemental Methods 1: Search strategy**

Pubmed:

*(((("urinary incontinence"[MeSH Terms] OR ("urinary"[All Fields] AND "incontinence"[All Fields]) OR "urinary incontinence"[All Fields] OR ("incontinence"[All Fields] AND "urinary"[All Fields]) OR "incontinence, urinary"[All Fields]) OR ("urinary incontinence"[MeSH Terms] OR ("urinary"[All Fields] AND "incontinence"[All Fields]) OR "urinary incontinence"[All Fields]) OR ("nocturnal enuresis"[MeSH Terms] OR ("nocturnal"[All Fields] AND "enuresis"[All Fields]) OR "nocturnal enuresis"[All Fields] OR ("incontinence"[All Fields] AND "nighttime"[All Fields] AND "urinary"[All Fields])) OR ("diurnal enuresis"[MeSH Terms] OR ("diurnal"[All Fields] AND "enuresis"[All Fields]) OR "diurnal enuresis"[All Fields] OR ("incontinence"[All Fields] AND "daytime"[All Fields] AND "urinary"[All Fields])) OR "stress incontinence"[All Fields] OR "urge incontinence"[All Fields] OR (mix[All Fields] AND incontinence[All Fields]) OR "urinary loss"[All Fields] OR "urinary leakage"[All Fields] OR (("urine"[Subheading] OR "urine"[All Fields] OR "urine"[MeSH Terms]) AND "leak"[All Fields])) AND (("mortality"[Subheading] OR "mortality"[All Fields] OR "mortality"[MeSH Terms]) OR ("death"[MeSH Terms] OR "death"[All Fields]) OR "fatal outcome"[All Fields] OR ("mortality"[Subheading] OR "mortality"[All Fields] OR "survival"[All Fields] OR "survival"[MeSH Terms]) OR ("vital statistics"[MeSH Terms] OR ("vital"[All Fields] AND "statistics"[All Fields]) OR "vital statistics"[All Fields]) OR "Life Expectancy"[All Fields] OR "Hospital Mortality"[All Fields] OR (vival[All Fields] AND Rate[All Fields]))))*

Embase / Cochrane

*'stress incontinence' OR 'urge incontinence'/exp OR 'urge incontinence' OR 'mix incontinence' OR 'urinary loss' OR 'urinary leakage'/exp OR 'urinary leakage' OR 'urine leak OR (urinary AND ('incontinence' OR 'incontinence'/exp OR incontinence)) AND 'mortality'/exp OR 'mortality' OR 'mortality'/exp OR mortality OR 'death'/exp OR 'death' OR 'death'/exp OR death OR 'fatal outcome'/exp OR 'fatal outcome' OR 'survival'/exp OR 'survival' OR 'survival'/exp OR survival OR vital AND ('statistics'/exp OR 'statistics' OR 'statistics'/exp OR statistics) OR 'life expectancy'/exp OR 'life expectancy' OR 'hospital mortality'/exp OR 'hospital mortality' OR 'vival rate'*

**Supplemental Methods 2: Assessment of global HR from different subgroups**

1. Pooled estimates for studies reporting HR by gender subgroups

- 2 studies (Thom 1997, Nuotio 2002) reported the ajusted HR by gender but not a global HR. To solve this issue, we assessed a global logarithm of HR as a weighted average of the gender-specific logarithm of HRs (with the inverse of the variance as weights, like a meta-analysis of logarithm of HRs with fixed effect). With this approach, these studies appeared only once in the forest plot. We obtained the following global HRs for these two studies:

Thom 1997: HR=1.158 (95%CI 1.083 to 1.239)

Nuotio 2002: HR=1.705 (95%CI 1.221 to 2.382)

1. Pooled estimates for studies reporting HR by UI severity subgroups

- 3 studies (Nakanishi 1999, Johnson 2000, Berardelli 2013) reported the ajusted HR by sub-group of severity of UI but did not report a global HR for UI, independently of the severity of UI. We also combined the HRs reported by severity of UI in a single global HR using a similar approach. However, in contrast to the gender-specific HRs, the severity-specific HRs from a study are not independent since the same patients (continent) are used to assess the HRs in each severity strata. Therefore, we pooled the severity-specific HRs assuming a correlation between the HRs. We used the function rma.mv of the R package meta for this purpose. To determine the correlation between the HRs, which is not reported in studies, we ran a Cox regression model with the patient-level data from the study by John et al. and we obtained a correlation coefficient around 0.20 between the estimates of the logarithm of HRs (from 0.17 to 0.24 for the multivariable model and from 0.18 to 0.20 for the univariate model). We verified the validity of our approach by comparing the re-assessed global HR and the global HR directly estimated with individual data from John study and results were similar:

Re-assessed global adjusted logarithm of HR: 0.5081 (se 0.2353)

Directly assessed logarithm of HR: 0.5128 (se 0.2130)

- We also compared the global unadjusted HR re-assessed from severity-specific HRs using this approach and the reported global unadjusted HR:

John 2014:

o   Re-assessed global non adjusted logarithm of HR: 0.7618 (se 0.1866)

o   Directly assessed non adjusted logarithm of HR : 0.7324 (se 0.1912)

Nakanishi 1999 :

o   Re-assessed global non adjusted logarithm of HR: 1.4317 (se 0.1691)

o   Directly assessed non adjusted logarithm of HR : 1.3051 (se 0.1555)

Herzog 1994 :

o   Re-assessed global non adjusted logarithm of HR: -0.1804 (se 0.1307)

o   Directly assessed non adjusted logarithm of HR : -0.1074 (se 0.1103)

Gavira 2005 :

o   Re-assessed global non adjusted logarithm of HR: 0.1193 (se 0.1519)

o   Directly assessed non adjusted logarithm of HR : 0.1196 (se 0.1512)

Johnson 2000 :

o   Re-assessed global non adjusted logarithm of HR: 0.5834 (se 0.0756)

o   Directly assessed non adjusted logarithm of HR : 0.5710 (se 0.0763)

- The obtained global HRs using our approach and the reported global HRs and their standard errors were similar. Therefore, we applied our approach to re-assess the global adjusted HRs, with a correlation coefficient of 0.20, the estimated adjusted global HRs were:

Nakanishi 1999: HR=1.436 (95%CI 0.974 to 2.118)

Johnson 2000: HR=1.086 (95%CI 0.809 to 1.458)

Berardelli 2013: HR=1.262 (95%CI 0.948 to 1.679)

- We performed a sensitivity analysis where we varied the coefficient of correlation from -0.20 to 0.70 to determine the global HR of the 3 studies (Nakanishi 1999, Johnson 2000, Berardelli 2013) and repeated a meta-analysis of adjusted models for each coefficient of correlation. The figure (below) shows that the variation of the coefficient of correlation has no impact on the final pooled HR.

With this approach, we avoided the repetition of studies in figure 4: we completed the method section, added a supplemental statistics (S2), and changed the figure 4 and table 2 accordingly.

**Supplemental Methods 3: Pooled HR ratios for UI severity**

1. Non adjusted HRs by severity of UI

For the subgroup analysis stratified by UI severity, we encountered the same problem exposed previously, that is the severity-specific HRs from a study are not independent since the same patients (continent) are used to assess the HRs in each severity strata. To analyse the increase of HR with the severity of UI, we assessed for each study the difference in logarithm of HRs between 2 stages of severity (assuming a correlation of 0.20) and we combined these differences. The results were expressed as the pooled ratio of HRs between two stages of severity of UI (exponential of the pooled difference in logarithm of HRs):

The ratio of HRs did not vary sensitively when the correlation coefficient ranged from -0.20 to 0.60 (figures below) and the confidence 95% interval contained the value 1 (equality of HRs) for any value of correlation coefficient (ratio between moderate and light UI and ratio between severe and moderate UI) or did not contain the value 1 (equality of HRs) for any value of correlation coefficient (ratio between severe and light UI). In conclusion, the interpretation of results was robust in regard of the correlation coefficient.


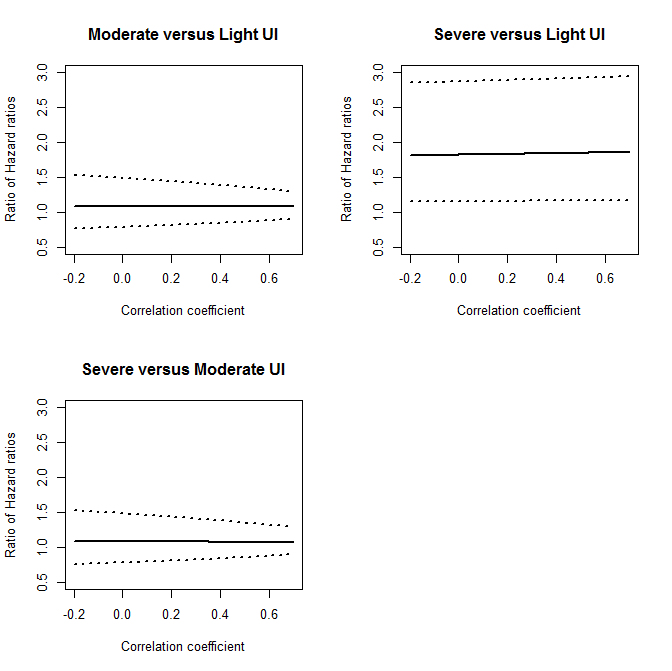


1. Adjusted HRs by severity of UI

As before, to analyse the increase of HR with the severity of UI, we assessed for each study the difference in logarithm of HRs between 2 stages of severity (assuming a correlation of 0.20) and we combined these differences. The results were expressed as the pooled ratio of HRs between two stages of severity of UI (exponential of the pooled difference in logarithm of HRs):

Interpretation of results was not modified when the value of the correlation coefficient ranged from -0.20 to 0.70.


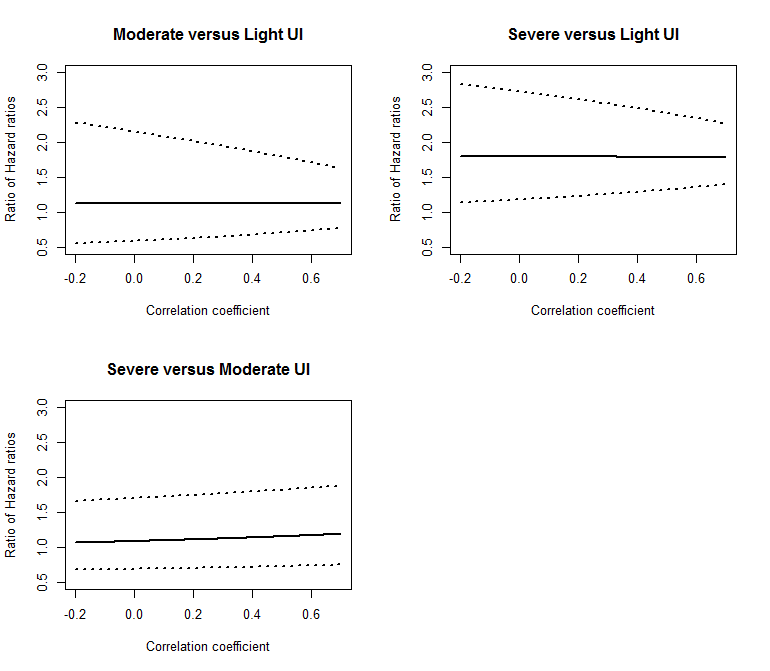

Supplement: S1 File — (DOCX) [file pone.0158992.s001.docx]
