# Supplementary material for: Urinary Incontinence as a Predictor of Death: A Systematic Review and Meta-Analysis
Source: PLoS One. 2016 Jul 13;11(7):e0158992. doi: 10.1371/journal.pone.0158992 (PMC4943733; doi:10.1371/journal.pone.0158992)
Supplement: S2 File — (DOCX) [file pone.0158992.s002.docx]

Supplemental material S2

Supplemental Table A Newcastle-Ottawa Quality Assessment scale p. 2

Supplemental Table B Adjustment variables included in multivariate models p. 3

Supplemental Table C Subgroup analyses stratified on the three domains of the Newcastle-Ottawa Quality Assessment scale p. 4

Supplemental Table D PRISMA check-list p.5-7

Supplemental Figure A Forest plot of adjusted OR of death at 6 months, 1, 3, 5, and 10 years p. 8

Supplemental Figure B Forest plot of unadjusted HR of death for urinary incontinence by frequencies of leakage episodes p. 9

Supplemental Figure C Forest plot of unadjusted difference of logarithm of HR between men and women p. 10

Supplemental Figure D Forest plot of adjusted HR of death for urinary incontinence by frequencies of leakage episodes p. 11

Supplemental Figure E Forest plot of adjusted difference of logarithm of HR between men and women p. 12

| **Supplemental Table A:** Newcastle-Ottawa Quality Assessment scale | | | | |
| --- | --- | --- | --- | --- |
| **Study** | **Selection** | **UI definition^*^** | **Compara**  **-bility** | **Outcome** |
| Abrahamik 1993 | 4 | 1 | 0 | 1 |
| Adams 2000 | 2 | 0 | 0 | 2 |
| Anpalahan 2008 | 3 | 1 | 1 | 2 |
| Baztan 2005 | 3 | 1 | 2 | 3 |
| Berardelli 2013 | 3 | 1 | 2 | 2 |
| Berrios 1986 | 2 | 0 | 0 | 3 |
| Bootsma 2013 | 2 | 0 | 2 | 3 |
| Brauer 1978 | 2 | 0 | 0 | 2 |
| Campbell (A And A) 1985 | 4 | 1 | 0 | 3 |
| Campbell (J epidemiol) 1985 | 4 | 1 | 2 | 3 |
| Chen 2010 | 3 | 1 | 0 | 3 |
| Donaldson 1983 | 2 | 0 | 2 | 3 |
| Donaldson 1980 | 2 | 0 | 0 | 3 |
| Ekelund 1987 | 3 | 1 | 0 | 3 |
| Espallargues 2008 | 3 | 0 | 2 | 1 |
| Gambassi 1999 | 3 | 1 | 2 | 2 |
| Gavira 2005 | 4 | 1 | 2 | 3 |
| Goldfarb 1969 | 2 | 0 | 0 | 1 |
| Herzog 1994 | 4 | 1 | 2 | 3 |
| Hollins 1998 | 2 | 0 | 0 | 3 |
| Holroyd-Leduc 2004 | 4 | 1 | 2 | 3 |
| John 2014 | 3 | 1 | 2 | 3 |
| Johnson 2000 | 4 | 1 | 2 | 3 |
| Kohn 1991 | 2 | 0 | 0 | 3 |
| Koyano 1986 | 3 | 0 | 0 | 3 |
| Krumholz 2001 | 2 | 0 | 2 | 3 |
| Landi 2012 | 4 | 1 | 2 | 3 |
| Luk 2013 | 2 | 0 | 0 | 3 |
| Min 2009 | 4 | 1 | 2 | 3 |
| Nakanishi 1999 | 4 | 1 | 2 | 3 |
| Nuotio 2009 | 2 | 0 | 2 | 3 |
| Nuotio 2002 | 2 | 0 | 2 | 3 |
| Pagliacci 2007 | 2 | 0 | 2 | 2 |
| Sorbye 2013 | 3 | 1 | 0 | 3 |
| Thom 1997 | 2 | 0 | 2 | 2 |
| Tilvis 1995 | 3 | 1 | 0 | 3 |
| Venkatsen 1990 | 2 | 0 | 0 | 2 |
| Zweig 1990 | 2 | 0 | 1 | 2 |
| ^*^ "Urinary incontinence (UI) definition" is one of the 4 items included in the "selection of study groups" domain of the Newcastle-Ottawa Quality Assessment scale. | | | | |

| **Supplemental Table B:** Adjustment variables included in multivariate models | | | | | | | | | |
| --- | --- | --- | --- | --- | --- | --- | --- | --- | --- |
| **Author** | **Age** | **Gender** | **Fct status** | **IUC** | **Cognition** | **Com-**  **orbid** | **Mood** | **Tobacco**  **/alcohol** | **BMI*** |
| Adams 2000 |  |  | **1** |  |  |  |  |  | **1** |
| Anpalahan 2008 |  |  |  |  |  | **1** |  |  |  |
| Baztan 2005 | **1** | **1** | **1** |  | **1** | **1** |  |  | **1** |
| Berardelli 2013 | **1** | **1** | **1** |  |  |  |  |  |  |
| Bootsma 2013 | **1** | **1** | **1** | **1** | **1** | **1** |  |  |  |
| Brauer 1978 |  | **1*** | **1*** |  |  |  |  |  |  |
| Campbell 1985 | **1** |  | **1** |  | **1** |  |  |  |  |
| Chen 2010 |  |  |  |  |  | **1** |  |  |  |
| Donaldson 1983 | **1** | **1** | **1** |  |  |  |  |  |  |
| Espalallargues 2008 | **1** | **1** | **1** |  | **1** | **1** |  |  |  |
| Gambassi 1999 | **1** | **1** | **1** |  | **1** | **1** |  |  |  |
| Gavira 2005 | **1** | **1** | **1** |  |  |  |  |  |  |
| Herzog 1994 | **1** |  | **1** |  |  | **1** |  |  |  |
| Hollins 1998 | **1** |  |  |  |  |  |  |  |  |
| Holroyd-Leduc 2004 | **1** | **1** | **1** |  | **1** | **1** | **1** | **1** | **1** |
| John 2014 | **1** | **1** | **1** |  | **1** | **1** | **1** | **1** | **1** |
| Johnson 2000 | **1** | **1** | **1** |  |  | **1** |  |  |  |
| Krumholz 2001 | **1** |  | **1** |  |  | **1** |  |  | **1** |
| Nakanishi 1999 | **1** | **1** | **1** |  |  | **1** | **1** |  |  |
| Nuotio 2002 | **1** | **1** | **1** |  |  | **1** | **1** |  |  |
| Nuotio 2009 | **1** | **1** | **1** |  |  | **1** |  | **1** |  |
| Pagliacci 2007 | **1** | **1** | **1** |  |  |  |  |  |  |
| Thom 1997 | **1** | **1**^†^ |  |  | **1** | **1** | **1** |  |  |
| Tilvis 1995 | **1** | **1** |  |  | **1** |  |  |  |  |
| Zweig 1990 |  |  | **1** |  | **1** | **1** |  |  |  |
| * Association between UI and death was stratified on gender and functional status.; † adjustment for BMI, weight loss or albuminemia;  Fct status: functional status; UICAT: presence of an urinary indwelling catheter, comorbide condition; BMI: body mass index. | | | | | | | | | |

| **Supplemental Table C: Subgroup analyses stratified on the three domains of the Newcastle-Ottawa Quality Assessment scale.** | | | | | | | | | | | | | | | | | | |
| --- | --- | --- | --- | --- | --- | --- | --- | --- | --- | --- | --- | --- | --- | --- | --- | --- | --- | --- |
|  | | **Unadjusted analysis** | | | | | | | | | | **Adjusted analysis** | | | | | | |
| **Factors** | | **N studies** | | | **Pooled HR** | | | **Within strata** | | **Between strata comparison** | | **N studies** | | **Pooled HR** | | **Within strata** | **Between strata comparison** | |
| ***"Selection" quality on the NOQ scale*** | | | | | | | | |  | |  | |  | |  | | |  |
| *Good or Fair* | 16 | | | 2.09 (1.65 to 2.64) | | | <0.0001 | | 0.60 | | 7 | | 1.17 (1.03 to 1.32) | | 0.0100 | | | 0.15 |
| *Low* | 12 | | | 2.34 (1.64 to 3.34) | | | <0.0001 | |  | | 7 | | 1.37 (1.14 to 1.63) | | 0.0006 | | |  |
| ***"Comparability" quality on the NOQ scale*** | | | |  | |  |  | |  | |  | |  | |  | | |  |
| *Good or Fair* | 14 | | | 1.85 (1.33 to 2.58) | | | 0.0003 | | 0.06 | | 10 | | 1.27 (1.10 to 1.48) | | 0.0004 | | | 0.87 |
| *Low* | 13 | | | 2.72 (2.19 to 3.36) | | | <0.0001 | |  | | 4 | | 1.24 (1.02 to 1.52) | | 0.0300 | | |  |
| ***"Outcome" quality on the NOQ scale*** | | |  | | |  |  | |  | |  | |  | |  | | |  |
| *Good or Fair* | 25 | | | 2.17 (1.70 to 2.76) | | | <0.0001 | | 0.46 | | 14 | | 1.27 (1.13 to 1.42) | | <0.0001 | | | - |
| *Low* | 2 | | | 2.45 (1.96 to 3.07) | | | <0.0001 | |  | | 0 | | - | | - | | |  |
| HR: hazard ratio; NOQ scale: Newcastle-Ottawa Quality Assessment scale | | | | | | | | | | | | | | | | | | |

**Supplemental Table D: PRISMA Checkliste**

| **Section/topic** | **#** | **Checklist item** | **Reported on page #** |
| --- | --- | --- | --- |
| **TITLE** | | |  |
| Title | 1 | Identify the report as a systematic review, meta-analysis, or both. | 1 |
| **ABSTRACT** | | |  |
| Structured summary | 2 | Provide a structured summary including, as applicable: background; objectives; data sources; study eligibility criteria, participants, and interventions; study appraisal and synthesis methods; results; limitations; conclusions and implications of key findings; systematic review registration number. | 2 |
| **INTRODUCTION** | | |  |
| Rationale | 3 | Describe the rationale for the review in the context of what is already known. | 3 |
| Objectives | 4 | Provide an explicit statement of questions being addressed with reference to participants, interventions, comparisons, outcomes, and study design (PICOS). | 3-4 |
| **METHODS** | | |  |
| Protocol and registration | 5 | Indicate if a review protocol exists, if and where it can be accessed (e.g., Web address), and, if available, provide registration information including registration number. | 4 |
| Eligibility criteria | 6 | Specify study characteristics (e.g., PICOS, length of follow-up) and report characteristics (e.g., years considered, language, publication status) used as criteria for eligibility, giving rationale. | 4-5 |
| Information sources | 7 | Describe all information sources (e.g., databases with dates of coverage, contact with study authors to identify additional studies) in the search and date last searched. | 4 |
| Search | 8 | Present full electronic search strategy for at least one database, including any limits used, such that it could be repeated. | appendix |
| Study selection | 9 | State the process for selecting studies (i.e., screening, eligibility, included in systematic review, and, if applicable, included in the meta-analysis). | 4-5 |
| Data collection process | 10 | Describe method of data extraction from reports (e.g., piloted forms, independently, in duplicate) and any processes for obtaining and confirming data from investigators. | 5 |
| Data items | 11 | List and define all variables for which data were sought (e.g., PICOS, funding sources) and any assumptions and simplifications made. | 5 |
| Risk of bias in individual studies | 12 | Describe methods used for assessing risk of bias of individual studies (including specification of whether this was done at the study or outcome level), and how this information is to be used in any data synthesis. | 5-appendix |
| Summary measures | 13 | State the principal summary measures (e.g., risk ratio, difference in means). | 6-7 |
| Synthesis of results | 14 | Describe the methods of handling data and combining results of studies, if done, including measures of consistency (e.g., I^2^) for each meta-analysis. | 6-8 |

Page 1 of 2

| **Section/topic** | **#** | **Checklist item** | **Reported on page #** |
| --- | --- | --- | --- |
| Risk of bias across studies | 15 | Specify any assessment of risk of bias that may affect the cumulative evidence (e.g., publication bias, selective reporting within studies). | 5-7 |
| Additional analyses | 16 | Describe methods of additional analyses (e.g., sensitivity or subgroup analyses, meta-regression), if done, indicating which were pre-specified. | 6-8 |
| **RESULTS** | | |  |
| Study selection | 17 | Give numbers of studies screened, assessed for eligibility, and included in the review, with reasons for exclusions at each stage, ideally with a flow diagram. | 8, Fig 1 |
| Study characteristics | 18 | For each study, present characteristics for which data were extracted (e.g., study size, PICOS, follow-up period) and provide the citations. | 8-12 tab1 |
| Risk of bias within studies | 19 | Present data on risk of bias of each study and, if available, any outcome level assessment (see item 12). | 15-16, sup Tab1 |
| Results of individual studies | 20 | For all outcomes considered (benefits or harms), present, for each study: (a) simple summary data for each intervention group (b) effect estimates and confidence intervals, ideally with a forest plot. | Fig2, fig4 |
| Synthesis of results | 21 | Present results of each meta-analysis done, including confidence intervals and measures of consistency. | 8-10, Fig2-5  Sup Fig1-5, table2 |
| Risk of bias across studies | 22 | Present results of any assessment of risk of bias across studies (see Item 15). | Fig6 |
| Additional analysis | 23 | Give results of additional analyses, if done (e.g., sensitivity or subgroup analyses, meta-regression [see Item 16]). | Tab2, sup fig3,5 |
| **DISCUSSION** | | |  |
| Summary of evidence | 24 | Summarize the main findings including the strength of evidence for each main outcome; consider their relevance to key groups (e.g., healthcare providers, users, and policy makers). | 20 |
| Limitations | 25 | Discuss limitations at study and outcome level (e.g., risk of bias), and at review-level (e.g., incomplete retrieval of identified research, reporting bias). | 22 |
| Conclusions | 26 | Provide a general interpretation of the results in the context of other evidence, and implications for future research. | 23 |
| **FUNDING** | | |  |
| Funding | 27 | Describe sources of funding for the systematic review and other support (e.g., supply of data); role of funders for the systematic review. | - |

*From:*  Moher D, Liberati A, Tetzlaff J, Altman DG, The PRISMA Group (2009). Preferred Reporting Items for Systematic Reviews and Meta-Analyses: The PRISMA Statement. PLoS Med 6(6): e1000097. doi:10.1371/journal.pmed1000097

For more information, visit: **www.prisma-statement.org**.

Page 2 of 2

**Supplemental Figure A:** Forest plot of unadjusted odds ratios (OR) of death for urinary incontinence. UI: urinary incontinence.


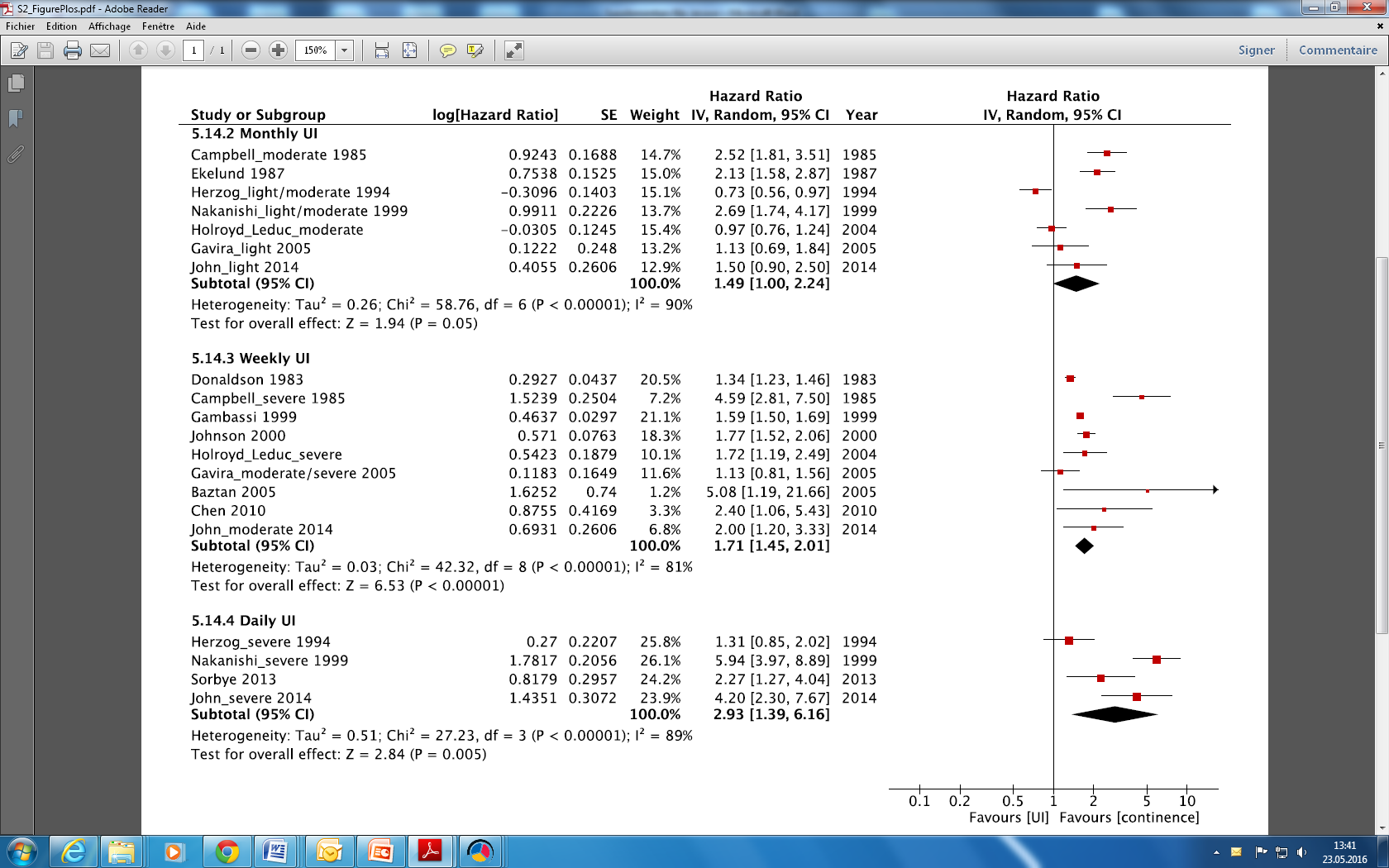


**Supplemental Figure B:** Forest plot of unadjusted HR of death for urinary incontinence by frequency of urinary leak. UI is classified depending on the minimum event per amount of time to be diagnosed. Thus some studies classified in yearly episodes might include patients with daily episodes. * Estimated from the ratio of logarithms of event-free proportions from the published proportion of death in the exposed and unexposed groups. † HR estimated from the Kaplan-Meier curve. UI: urinary incontinence. UI: urinary incontinence.

**Supplemental Figure C:** Forest plot of pooled unadjusted difference in logarithm of HR between men and women. * Estimated from the ratio of logarithms of event-free proportions from the published proportion of death in the exposed and unexposed groups. † HR estimated from the Kaplan-Meier curve. UI: urinary incontinence. UI: urinary incontinence.


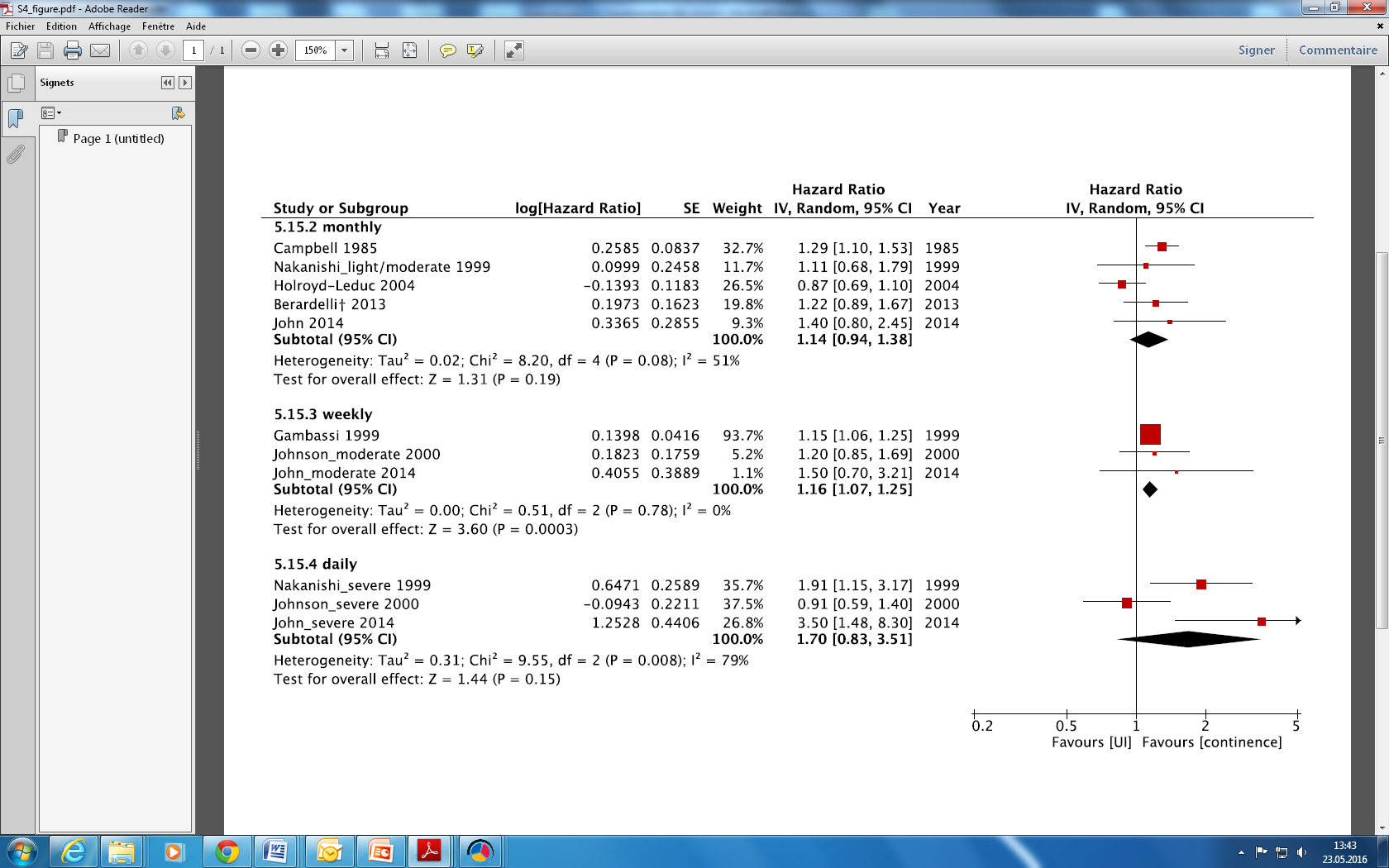


**Supplemental Figure D:** Forest plot of adjusted HR of death for urinary incontinence by frequency of urinary leak. UI is classified depending on the minimum event per amount of time to be diagnosed. Thus some studies classified in yearly episodes might include patients with daily episodes. UI: urinary incontinence.

**Supplemental Figure E:** Forest plot of pooled adjusted difference in logarithm of HR between men and women. * Estimated from the ratio of logarithms of event-free proportions from the published proportion of death in the exposed and unexposed groups. † HR estimated from the Kaplan-Meier curve. UI: urinary
